# A model for preservation of thymocyte-depleted thymus

**DOI:** 10.1590/1414-431X2023e12647

**Published:** 2023-08-14

**Authors:** A.S. Dias, N.R. Damaceno-Rodrigues, T.M. Gimenez, P.M. Oliveira, M.C. Zerbini, M. Carneiro-Sampaio, V. Odone, M.B. Jatene, D.M. Vasconcelos, V. Rocha, E.M. Novak

**Affiliations:** 1 Laboratório de Pediatria Clínica LIM36, Instituto da Criança, Hospital das Clínicas, Faculdade de Medicina, Universidade de São Paulo, São Paulo, SP, Brasil; 2 Instituto de Tratamento de Câncer Infantil, Instituto da Criança, Hospital das Clínicas, Faculdade de Medicina, Universidade de São Paulo, São Paulo, SP, Brasil; 3 Departamento de Patologia, Laboratório de Biologia Celular (LIM 59), Faculdade de Medicina, Universidade de São Paulo, São Paulo, SP, Brasil; 4 Setor de Cirurgia Cardíaca Pediátrica, Hospital do Coração da Associação do Beneficente Síria, São Paulo, SP, Brasil; 5 Departamento de Patologia, Faculdade de Medicina, Universidade de São Paulo, São Paulo, SP, Brasil; 6 Laboratório de Investigação Médica em Dermatologia e Imunodeficiências (LIM 56), Hospital das Clínicas, Faculdade de Medicina, Universidade de São Paulo, São Paulo, SP, Brasil; 7 Fundação Pró-Sangue São Paulo, Hemocentro de São Paulo, São Paulo, SP, Brasil; 8 Laboratório de Investigação Médica em Patogênese e Terapia dirigida em Onco-Imuno-Hematologia (LIM 31), Serviço de Hematologia, Hemoterapia e Terapia Celular, Hospital das Clínicas, Faculdade de Medicina, Universidade de São Paulo, São Paulo, SP, Brasil

**Keywords:** Thymus tissue, Depletion, Cryopreservation, DiGeorge syndrome

## Abstract

DiGeorge syndrome is a disorder caused by a microdeletion on the long arm of chromosome 22. Approximately 1% of patients diagnosed with DiGeorge syndrome may have an absence of a functional thymus, which characterizes the complete form of the syndrome. These patients require urgent treatment to reconstitute T cell immunity. Thymus transplantation is a promising investigational procedure for reconstitution of thymic function in infants with congenital athymia. Here, we demonstrate a possible optimization of the preparation of thymus slices for transplantation through prior depletion of thymocytes and leukocyte cell lineages followed by cryopreservation with cryoprotective media (5% dextran FP 40, 5% Me2SO, and 5% FBS) while preserving tissue architecture. Thymus fragments were stored in liquid nitrogen at -196°C for 30 days or one year. The tissue architecture of the fragments was preserved, including the distinction between medullary thymic epithelial cells (TECs), cortical TECs, and Hassall bodies. Moreover, depleted thymus fragments cryopreserved for one year were recolonized by intrathymic injections of 3×10^6^ thymocytes per mL, demonstrating the capability of these fragments to support T cell development. Thus, this technique opens up the possibility of freezing and storing large volumes of thymus tissue for immediate transplantation into patients with DiGeorge syndrome or atypical (Omenn-like) phenotype.

## Introduction

DiGeorge syndrome (DGS) is the most common of the microdeletion syndromes. It occurs in approximately 1:4000 births and affects multiple organs, including the heart, the nervous system, and the kidney (1,2). The DGS phenotype is very heterogeneous with variable expression of the different features including immunodeficiency (3). The thymus gland supports the differentiation and selection of T cells. Thymus tissue removed from infants during cardiac surgery is discarded and can be used for immune system reconstitution to correct maturation of T cells in patients with DGS through thymus tissue transplantation (4). According to a previous study, thymus transplantation resulted in the survival of over 70% of athymic DGS patients (5,6). Moreover, patients with atypical (Omenn-like) phenotype that causes athymia can also be treated with thymus transplantation (7).

The thymus comprises thymic epithelial cells (TEC), mesenchymal cells, fibroblasts, dendritic cells, macrophages, and vascular endothelial cells. TECs are separated into cortical (cTECs) and medullary (mTECs) TECs, which constitute two functionally distinct microenvironments for T cell development (8).

Previously, Ross et al. (9) showed that the transplantation of cryopreserved and subsequently thawed human thymic tissue into athymic mice could support thymopoiesis, achieving reconstitution of T cells. However, thymus samples are not always available for use at the time of transplantation; in addition, the preparation of the thymic fragments requires an extensive 21-day culture period.

Here, we demonstrate a possible optimization of the preparation of thymus slices for transplantation while preserving tissue architecture, including the distinction between mTECs, cTECs, and Hassall's corpuscles.

## Material and Methods

### Thymic tissue preparation

Thirty-five thymus tissue samples from infant donors who underwent thymectomy were prepared as described by Hong and Moore (10). Donor age ranged between one and six months with a median age of four months.

Thymus tissue samples were washed with Ham's F-12 culture medium supplemented with 2 mM L-glutamine (Ham; Gibco, USA), 10% fetal bovine serum (FBS) (Gibco), 25 mM HEPES (Gibco), 100 µg/mL streptomycin sulfate (Gibco), 1 µg/mL gentamicin (Gibco), and 100 µg/mL of antifungal agent amphotericin B (Sigma-Aldrich, USA) (hereafter called supplemented medium), and decapsulated. Sixty 0.5-mm thick tissue slices measuring 15×15 mm were cut using a Stadie-Riggs tissue slicer (Thomas Scientific^©^, USA). Of these tissue slices, 15 thymus fragments that were not depleted of leukocytes and thymocytes were used as controls (CTRL), 15 thymus fragments were depleted of leukocytes and thymocytes for 21 days (DT), and 30 thymus fragments were depleted of leukocytes and thymocytes for 21 days followed by cryopreservation in liquid nitrogen at −196°C for 30 days (30-day DCT) or one year (one-year DCT) and thawing. All experimental procedures were performed under sterile conditions in a laminar flow hood. Thymic fragments selected for depletion were placed on a polymeric membrane in a culture plate containing Ham's F-12 culture medium supplemented with 1.34 mM deoxyguanosine (Sigma-Aldrich). Thymic fragments were incubated at 37°C at 5% CO_2_ for 21 days to complete depletion of thymocytes. The medium was changed every two days over the 21-day culture period.

### Cryopreservation and defrosting of human thymus tissue

Thirty depleted thymic fragments (DCT) were frozen in cryoprotective media containing Ham's F-12 culture supplemented medium with 2 mM L-glutamine and 5% Dextran FP 40, 5% Me_2_SO, and 5% FBS. Freezing was performed gradually in a cryocooler-based control-rate freezer at a rate of −1°C/min until reaching −100°C as described by Ross et al. (9). To allow time for ice nucleation and for dissipation of latent heat of ice formation, the following freezing program was used: −1°C/min to −10°C, hold at −10°C for 10 min, −1°C/min to −40°C, −1°C/min to −100°C. The frozen depleted thymic fragments were stored in liquid nitrogen at −196°C for either 30 days (15 samples) or one year (15 samples).

After storage, thymic fragments were thawed in 5 mL of Ham's F-12 culture medium supplemented with 5% FBS for 10 min at 37°C or until ice had disappeared. After defrosting, thymic fragments were washed twice in supplemented medium for 10 min.

For recolonization, thymic fragments cryopreserved for one year (one-year DCT) were transferred to a six-well culture plate with 3 mL of supplemented medium and placed in an oven at 37°C with 5% CO_2_ until use.

### Histological and immunohistochemistry analysis

Human thymus tissue was fixed in formalin and paraffin-embedded. Tissue was sectioned (5 μm) and evaluated by histological analysis using hematoxylin-eosin (H&E) staining and immunohistochemistry analysis using anti-CKPan (1:150; clone AE1/AE3, ThermoFisher Scientific, USA) antibody as described by Ross et al. (9).

### Flow cytometry of thymic samples

Thymus tissue samples (CTRL, DT, and DCT) were mechanically and enzymatically dissociated using collagenase I (2 mg/mL; Sigma-Aldrich, C0130) and DNase I (80 μg/mL; Roche, Switzerland) in RPMI 1640 (Gibco) and 2% FBS as described by Shichkin et al. (11). At the end of digestion, the cell suspension was passed through a cell filter (100 μm) and then stained with antibodies for flow cytometry analysis. Cells were stained with fluorochrome-conjugated antibodies in PBS with 5% FBS and 0.01% azide for 15 min at room temperature, as described by Ross et al. (9). To identify TEC populations, a phenotypic panel of labeled antibodies was used: CD326 APC (clone 9C4) and HLA-DR BV421 (clone G46-6), (BD Biosciences, USA), as described by Davies (3). To assess the loss of leukocyte and thymocyte lineage cells, CD45 Alexa Fluor 700 (clone HI 30) and CD3 PERCP (clone UCHT1) (BD Biosciences) were used, respectively.

Samples were analyzed by flow cytometry (LRS II Fortessa flow cytometer, BD Biosciences; software: FlowJo, Tree Star, USA). The gating strategy is shown in Figures 2 and 3 and Supplementary Figures S1 and S2. The gating was based on unstained controls for each antibody. Unstained controls are shown on the left side of the figures.

### Thymic tissue recolonization with thymocyte injections in one-year DCT

For thymic recolonization, 15 one-year DCT were treated with intrathymic (*it*) injections containing 3×10^6^ thymocytes per mL (TR). Thymocytes were obtained from fresh human thymus treated by enzymatic digestion (dissociation method) using collagenase I (2 mg/mL) and DNase I (80 μg/mL) in RPMI 1640 supplemented with 2% FBS as described by Shichkin et al. (11). Cells were then immediately resuspended in RPMI 1640, transferred to a 50-mL conical tube containing Ficoll-Paque (GE Healthcare Bio-Sciences, Sweden), and centrifuged at 540 *g* for 20 min at room temperature. Cells were washed twice with RPMI 1640 and centrifuged at 540 *g* for 10 min to 4°C. After separation, cells were adjusted to a concentration of 3×10^6^ thymocytes per mL and injected *it* in the right and left thymic lobes of one-year DCT samples, which had been previously defrosted as described above. Thymic fragments were incubated with 5 mL of Ham's F-12 culture medium supplemented with 5% FBS for 10 days in 5% CO_2_ at 37°C_._

To evaluate the efficiency of thymocyte recolonization, seven thymic fragments recolonized with thymocytes were fixed in formalin, paraffin-embedded, and stained with CD3+ antibody (clone UCHT1, ThermoFisher Scientific).

Additionally, to assess cell death of CD3+ T cells and CD4+, CD8+, double positive (CD4+CD8+) and double negative (CD4−CD8−) subpopulations, cells were labeled with fluorescence-conjugated antibodies (CD3 PERCP, clone UCHT1; CD4 Alexa fluorine 488, clone RPA-T4; CD8 APCH-7, clone SK1 (BD Biosciences)), stained with 7-aminoactinomycin D (7-AAD; BD Biosciences) and quantified by flow cytometry before recolonization of thymic fragments.

### Statistical analysis

For all experiments, the sample size (n) for each experimental group/condition is provided in the figure legends. Data were analyzed using nonparametric two-way analysis of variance (ANOVA) followed by the Newman-Keuls multiple comparison test. Plots and graphs were generated with GraphPad Prism 8.0 software (GraphPad Inc., USA).

## Results

**Figure 1:**
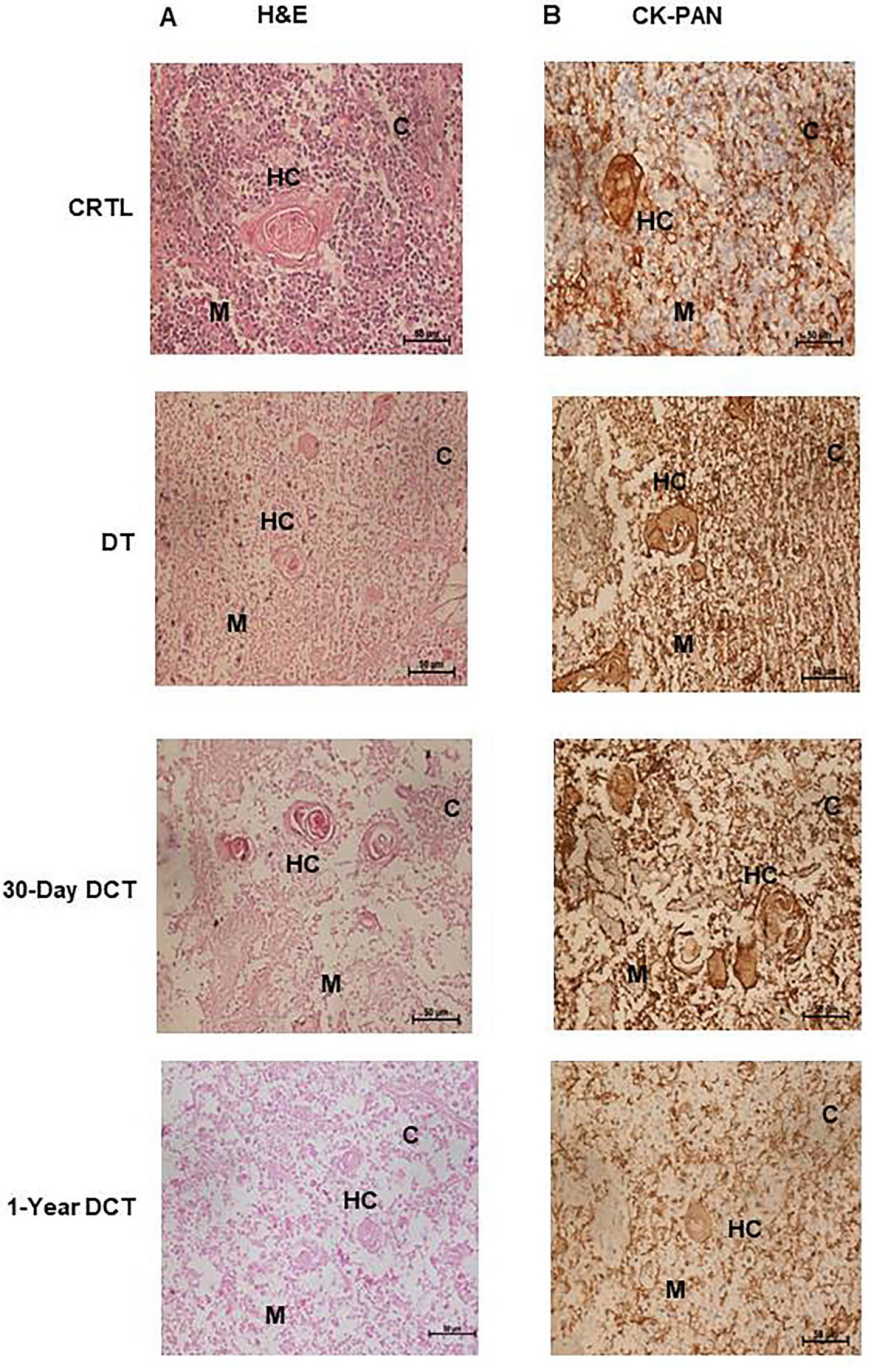
Histological (H&E) (**A**) and immunohistochemical (CK-PAN) (**B**) analysis of formalin-fixed paraffin-embedded fresh thymus tissue (CTRL), thymus tissue depleted for 21 days (DT), thymus tissue depleted for 21 days and cryopreserved (DCT). C, cortex, M: medulla, HC: Hassall's corpuscles. **A**, CTRL slices with basophilic thymic cortex densely packed with small and immature thymocytes, and lightly eosinophilic medulla due to the reduced number of thymocytes. Typical eosinophilic Hassall's corpuscles are observed. DT and DCT cortex lost their basophilia due to loss and karyolysis of thymocytes during the 21-day culture period or storage in liquid nitrogen/thawing for 30 days and one year. The integrity of thymic epithelium (cortex and medulla) and Hassall's corpuscles was observed. **B**, Immunohistochemical analysis with a pan-cytokeratin antibody (CKPan) staining of epithelial components revealing preserved tissue architecture of the thymus in CTRL, DT, and DCT samples. Brown color indicates a positive reaction. Scale bars: 50 µm.

Typical histologic features of the normal thymus including thymic cortex, composed of basophilic thymocytes and eosinophilic thymic medulla, and Hassall's corpuscles were observed in fresh human HE-stained thymus slices. DT and DCT samples showed thymic epithelium integrity and eosinophilic discoloration due to thymocytes lost from the tissue after the 21-day culture period or dead thymocytes with nuclear karyolysis showing well defined and eosinophilic Hassall's corpuscles (Figure 1).

Medullary epithelial and cortical epithelial cells were analyzed with anti-CKPan (pan-cytokeratin). Cytokeratin-positive cells were detected in CTRL, DT, and DCT samples. Immunohistochemistry analysis of these markers revealed preserved thymic architecture in CTRL, DT, and DCT samples (Figure 1).

**Figure 2:**
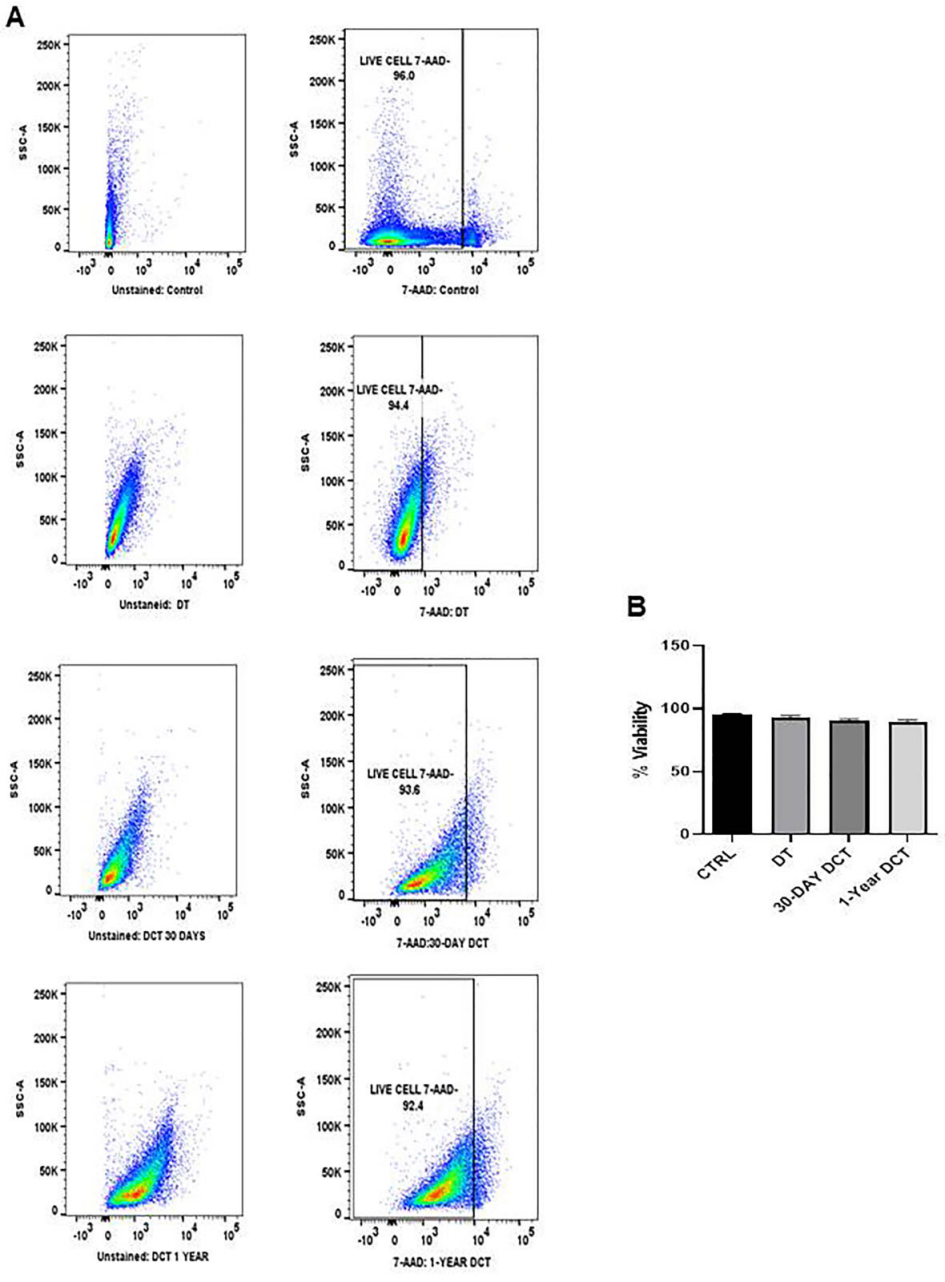
**A**, Histograms representative of cell viability (n=15) as measured by flow cytometry with 7-aminoactinomycin D (7-AAD) labeling at different time points of the protocol: fresh thymus tissue (CTRL), thymus tissue depleted for 21 days (DT), thymus tissue depleted for 21 days and cryopreserved for 30 days (30-day DCT), and thymus tissue depleted for 21 days and cryopreserved for one year (one-year DCT). Unstained controls are shown on the left side of the figure. **B**, Cell viability at different time points as assessed by 7-AAD staining. Data were analyzed using two-way analysis of variance (ANOVA) followed by the Newman-Keuls multiple comparison test. There were no significant differences between groups compared to CTRL (P>0.05). Data are reported as means±SE from 15 samples.

The degree of viability of thymic epithelial cells is essential in long-term thymus tissue cultures. Thus, the occurrence of cell death was assessed by 7-aminoactinomycin D (7-AAD) staining as described by Shichkin et al. (11). Cell viability was 96% in CTRL, 94.4% in DT, 93.6% in 30-day DCT, and 92.4% in one-year DCT samples (Figure 2A). Results showed that TEC viability in DT and DCT samples preserved in cryoprotective media was not affected after the depletion period or long-term storage in liquid nitrogen. In addition, there was no significant difference in cell viability between groups compared to CTRL (P>0.05, Figure 2B). Thus, the cryopreservation method used (as described in the Material and Methods section) was successful in preventing cell viability loss after thawing.

**Figure 3:**
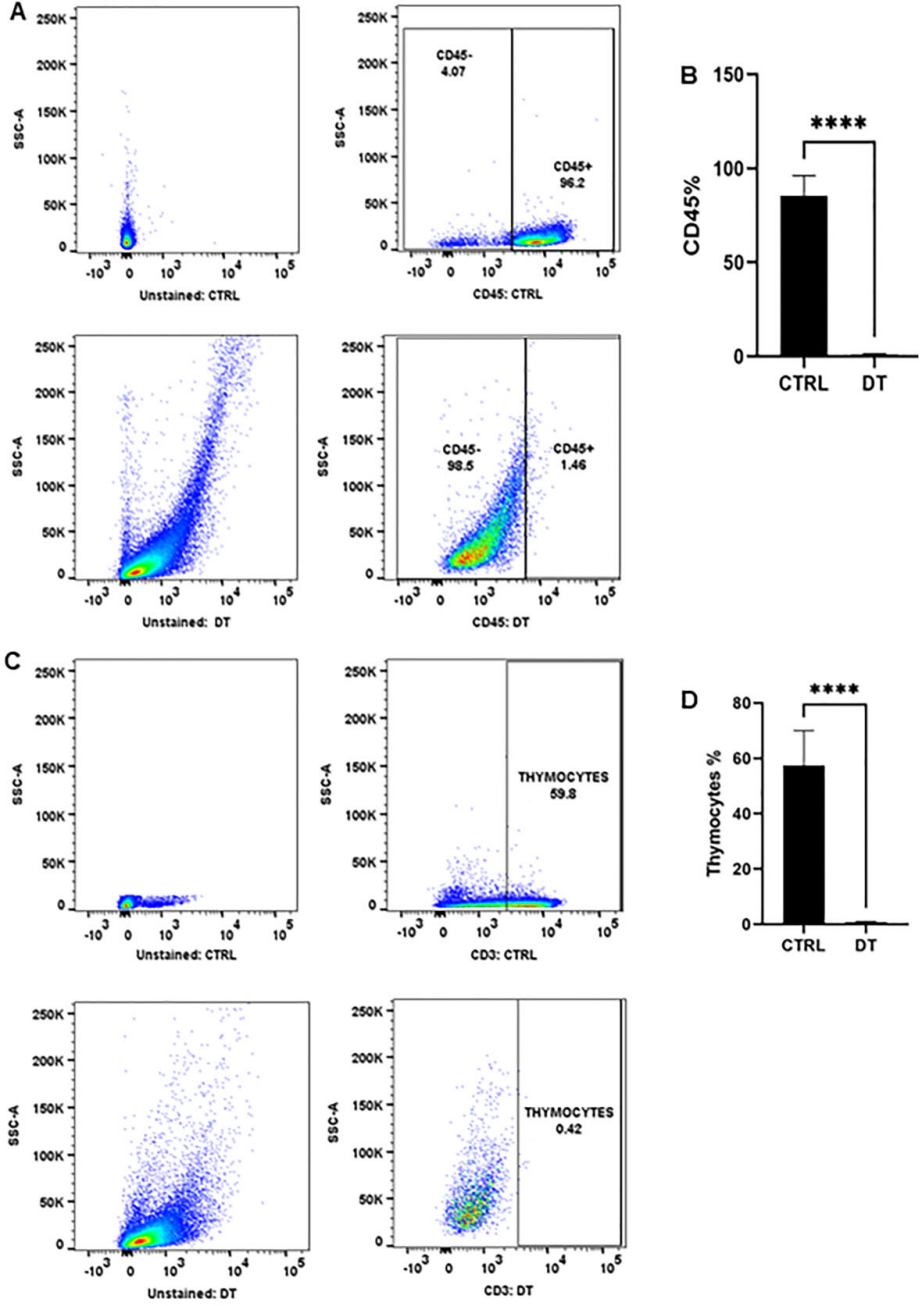
Analysis of leukocyte and thymocyte populations after a 21-day thymus tissue depletion period (DT). Fresh thymus tissue not depleted of leukocyte cells and thymocytes was used as control (CTRL). **A** and **C**, Gating strategy based on leukocyte marker CD45+ and thymocyte marker CD3+. Percentages and total numbers for each cell subset were quantified by flow cytometry. **B** and **D**, Percentages of CD45+ and CD3+ populations in DT compared to CTRL. Data are reported as means±SE for n=15. ****P<0.001, Newman-Keuls multiple comparison test. Unstained controls are shown on the left side of the figure.

Depletion of the thymus tissue was evaluated by loss of expression of CD45+ leukocytes and CD3+ thymocytes in CTRL and DT using the standard procedure of enzymatic dissociation of thymic tissue followed by flow cytometry analysis as described by Shichkin et al. (11). Leukocyte lineage CD45+ expression was 96.2% in CTRL, and after the 21-day thymic tissue depletion period, thymic stroma showed a significantly decreased leukocyte cell population (1.46%) compared to CTRL (P≤0.001) (Figure 3A and B).

The CD3+ thymocyte cell population reached 59.8% in CTRL but significantly declined to 0.41% after the 21-day tissue depletion period (P≤0.001) (Figure 3C and D). These results showed that thymus tissue fragments were successfully depleted of leukocyte cell lineages and thymocytes.

To compare TEC populations between 30-day and 1-year DCT samples, using DT samples as a control, we used a phenotypic panel for TEC (CD326+ HLA-DR+) as shown in Supplementary Figure S1A. Thymus tissues were prepared as described by Shichkin et al. (11).

The percentage of TECs expressing CD326+ HLA-DR+ was 96.9% in 30-day DCT and 94.3% in one-year DCT samples. There was no significant difference in TEC population between 30-day DCT and 1-year DCT compared to DT 97.4% (P>0.05, Supplementary Figure S1B).

To assess the functional potential of cryopreserved thymus fragments, 15 depleted thymus fragments cryopreserved for one year were recolonized with *it* injections of 3×10^6^ CD3+ thymocytes per mL in right and left thymic lobes as described in Materials and Methods. To evaluate the recolonization efficiency and thymocyte cell survival, thymus tissue slices were analyzed by immunohistochemical CD3 staining and thymocyte viability was evaluated in recolonized thymus fragments with 7-AAD staining by flow cytometry in thymocytes cells before recolonization used as control and after 10 days of thymocyte culture in one-year DCT (TR). Anti-CD3 antibody staining confirmed the presence of T cells in one-year DCT samples after 10 days of culture (TRCD3+) (Supplementary Figure S2A), suggesting that recolonization had no effect on thymocyte viability. The frequency of CD4 and CD8 thymocytes in one-year DCT samples was evaluated before and after recolonization. The frequency of thymocyte stages before recolonization was CD4+ 26.4%, CD8+ 10.2%, double-positive (DP) thymocytes 59.6%, and double-negative (DN) thymocytes 3.84% (Supplementary Figure S2B), but on day 10 of recolonization their frequency was: CD4+ 29.8%, CD8+ 14.2%, DP 53.0%, and DN 3.05% (Supplementary Figure S2C). These results showed an increase in frequency of CD4^+^ and CD8^+^ mature thymocytes/lymphocytes and a decrease of immature cell profile, suggesting that an extensive positive selection of CD4+ or CD8+ mature cells were formed after thymus tissue recolonization.

## Discussion

Thymus transplantation is indicated for the treatment of athymia and associated T cell deficiency in patients with DiGeorge syndrome, atypical (Omenn-like) phenotype, and *FOXN1* deficiency (4,7,12). Human thymic allotransplantation is performed using neonatal thymus tissue harvested routinely at the time of cardiovascular surgery for repair of congenital heart defects (4). The supply of such tissue, however, is obviously limited. Nevertheless, the use of cryopreserved thymus slices previously depleted of thymocytes and leukocyte cell lineages preserved in a biobank for transplantation of thymus tissue may make the treatment more readily available in the future and potentially increase the possibility for patients to be treated at institutions in their own country. Cryopreservation of such thick pieces of tissue on the scale required for clinical use is challenging because of choice of cryoprotectants, cooling protocols, and the control of ice nucleation (9). These complex events all impact viability of thawed tissue.

In this study, we demonstrated that previously decellularized thymus scaffolds preserved in cryoprotective media containing 5% Dextran FP40 (non-penetrating compound), 5% Me_2_SO (penetrating compound), and 5% FBS can support the survival of medullary epithelial cells and cortical epithelial cells with functional thymic epithelial cell architecture for thymic maturation. Structural features of the tissue such as cortex and medullary regions as well as Hassall’s corpuscles were identifiable via hematoxylin-eosin staining, whereas cytokeratin immunohistochemistry revealed that tissue architecture of the cryopreserved slices was similar to that of control fresh human thymus slices. A normal lacy pattern was found in both slices that had been frozen and control slices, again suggesting that the freezing process did not disrupt the architecture of the epithelial cells that constitute the thymic stroma.

The previously depleted thymus tissue also showed a decrease in CD45+ leukocytes lineage and especially in CD3+ thymocytes. CD45 is expressed differentially in T cells at different stages of development and activation (13), whereas CD3 is expressed on the membrane surface of thymocytes/T lymphocytes (13). The absence of leukocytes and thymocytes in the thymic tissue has significant implications for future thymus transplantation due to the potential to reduce graft rejection and prevent complications associated with the recognition of transplanted tissue by the patient's immune system cells (graft/host rejection) (4,14,15).

The cryoprotective solution was effective in maintaining cell viability after freezing for 30 days and one year at levels similar to controls, as evidenced by negative 7AA-D labeling in flow cytometry analysis (Figure 2). 7AA-D is a membrane-impermeable dye that is generally absent in viable cells, indicating that the cryopreservation method used in this study was able to successfully prevent cell death that occurs after the freezing-thawing procedure.

The thymus provides multiple microenvironments that are essential for the development and repertoire selection of T lymphocytes. Cortical and medullary TECs constitute the major stromal cells that structurally form and functionally characterize the cortex and the medulla, respectively (16). In the current study, phenotypic marking with anti-CD326 revealed high frequencies of epithelial populations in thymic fragments after cell depletion and cryopreservation for 30 days and one year, suggesting the remaining TEC populations remained functionally active during long-term storage in liquid nitrogen.

The functional potential of cryopreserved thymus fragments was also observed *in vitro* in thymus fragments cryopreserved for one year (DCT) and recolonized with CD3+ thymocytes. A significant increase in the frequency of CD4+ and CD8+ mature thymocytes/lymphocytes and a decrease in the profile of immature cells were observed in these fragments, suggesting extensive positive selection for CD4+ or CD8+ mature cells after thymus tissue recolonization.

In summary, thymocyte recolonization in one-year DCT samples demonstrated the capacity to support thymocyte differentiation and contribute with significant numbers of precursors to T cell development.

Additional studies using nude mice transplanted (9) with thymus fragments cryopreserved for 30-days and one-year as described in this study will be necessary to answer some questions about potential limitations of the protocol that may affect the success of thymus transplantation, for example, whether transplantation of thymic slices results in production of mouse T cells expressing CD4, CD8, and CD3 or whether long-term cryopreservation of thymus fragments increases the risk of rejection of the transplanted thymus through increased DAMPs (damage-associated molecular patterns) release and activation of donor antigen-presenting cells (APCs) (17).

This preliminary *in vitro* study showed that cryoprotective media designed specifically for freezing thymus slices for long-term storage in liquid nitrogen preserved TEC architecture and function to support T-lymphocyte development. Thus, this technique opens up the possibility of freezing and storing thymus tissue for immediate transplantation into patients with DiGeorge syndrome, *FOXN1* deficiency, and atypical (Omenn-like) phenotype.

## Supplementary Material

Click here to view [pdf].
